# The oxidative burst reaction in mammalian cells depends on gravity

**DOI:** 10.1186/1478-811X-11-98

**Published:** 2013-12-20

**Authors:** Astrid Adrian, Kathrin Schoppmann, Juri Sromicki, Sonja Brungs, Melanie von der Wiesche, Bertold Hock, Waldemar Kolanus, Ruth Hemmersbach, Oliver Ullrich

**Affiliations:** 1 Department of Machine Design, Engineering Design and Product Development, Institute of Mechanical Engineering, Otto-von-Guericke-University Magdeburg, Universitätsplatz 2, D-39106 Magdeburg, Germany; 2 Institute of Anatomy, Faculty of Medicine, University of Zurich, Winterthurerstrasse 190, CH-8057 Zurich, Switzerland; 3 Gravitational Biology, Biomedical Science Support Center, Institute of Aerospace Medicine, German Aerospace Center (DLR), Linder Hoehe, 51147 Koeln, Germany; 4 EADS Astrium, TEB 22 Payload Engineering, Claude Dornier Strasse, 88039 Friedrichshafen, Germany; 5 Department Biology II, LMU München, Großhaderner Strasse 2, 82152 Planegg-Martinsried, Germany; 6 Study Team SAHC, Institute of Aerospace Medicine, German Aerospace Center (DLR), Linder Hoehe, 51147 Koeln, Germany; 7 Chair of Proteomics and Bioanalytics, TU München, Alte Akademie 10, 85354, Freising, Germany; 8 Laboratory of Molecular Immunology, Life and Medical Sciences (LIMES) Institute, University of Bonn, Carl-Troll-Strasse 31, 53115, Bonn, Germany; 9 Study Group “Magdeburger Arbeitsgemeinschaft für Forschung unter Raumfahrt- und Schwerelosigkeitsbedingungen” (MARS), Otto-von-Guericke-University Magdeburg, Universitätsplatz 2, D- 39106, Magdeburg, Germany; 10 Zurich Center for Integrative Human Physiology (ZIHP), University of Zurich, Zurich, Switzerland

**Keywords:** Microgravity, Hypergravity, Clinorotation, Parabolic flight, Macrophage, Oxidative burst, Phagocytosis

## Abstract

Gravity has been a constant force throughout the Earth’s evolutionary history. Thus, one of the fundamental biological questions is if and how complex cellular and molecular functions of life on Earth require gravity. In this study, we investigated the influence of gravity on the oxidative burst reaction in macrophages, one of the key elements in innate immune response and cellular signaling. An important step is the production of superoxide by the NADPH oxidase, which is rapidly converted to H_2_O_2_ by spontaneous and enzymatic dismutation. The phagozytosis-mediated oxidative burst under altered gravity conditions was studied in NR8383 rat alveolar macrophages by means of a luminol assay. Ground-based experiments in “functional weightlessness” were performed using a 2 D clinostat combined with a photomultiplier (PMT clinostat). The same technical set-up was used during the 13th DLR and 51st ESA parabolic flight campaign. Furthermore, hypergravity conditions were provided by using the Multi-Sample Incubation Centrifuge (MuSIC) and the Short Arm Human Centrifuge (SAHC). The results demonstrate that release of reactive oxygen species (ROS) during the oxidative burst reaction depends greatly on gravity conditions. ROS release is 1.) reduced in microgravity, 2.) enhanced in hypergravity and 3.) responds rapidly and reversible to altered gravity within seconds. We substantiated the effect of altered gravity on oxidative burst reaction in two independent experimental systems, parabolic flights and 2D clinostat / centrifuge experiments. Furthermore, the results obtained in simulated microgravity (2D clinorotation experiments) were proven by experiments in real microgravity as in both cases a pronounced reduction in ROS was observed. Our experiments indicate that gravity-sensitive steps are located both in the initial activation pathways and in the final oxidative burst reaction itself, which could be explained by the role of cytoskeletal dynamics in the assembly and function of the NADPH oxidase complex.

## Introduction

A variety of gravity-sensing mechanisms evolved in multicellular and complex organisms to benefit from this constant force for orientation in three-dimensional space. However, the gravitational force has also been shown to affect cellular and molecular systems in mammalian cells. Since the 1980’s, considerable evidence has been obtained showing that mammalian cells and small unicellular organisms function differently under the conditions of microgravity [1-4]. This led to the question of how gravitational force might play a role in cellular function and whether gravity might provide important signals for the cell. Due to the fact that cells of the human immune system are sensitive to altered gravity, they present a well-suited biological model system in investigating whether the Earth’s gravity is important for signal transduction processes in mammalian cells and investigating basic molecular and cellular mechanisms of gravi-sensitivity [5,6].

The monocyte-macrophage-system (MMS) belongs to the innate immune system and represents the body’s first line of defense. The innate immune system is characterized by a fast but unspecific immune reaction and activates the adaptive immune response. This is done mainly through interaction of antigen-presenting cells (APCs) and dendritic cells, but also macrophages [7] with T lymphocytes. Monocytes circulate in the blood stream, enter tissues and differentiate into their mature form: the macrophages [7]. Macrophages are relatively long-lived, carry a variety of surface receptors, such as pattern recognition receptors and receptors for antibodies and complement components. They reside in tissues in the whole body, e.g. the intestinal tract, the respiratory tract, the liver, the spleen, the bone, and connective tissues [8]. During the progress of phagocytosis after pattern recognition, an arsenal of killing agents is released, which includes the assembly of NADPH oxidase complexes on the phagolysosomal membranes. This catalyzes the production of oxygen-derived, highly toxic compounds, e.g. superoxide (O_2_^-^), hypochloride (HOCl), hydroxyl radicals or hydrogen peroxide (H_2_O_2_), a process which is known as the oxidative burst [8]. Reactive oxygen species, especially H_2_O_2_, may also be involved in signaling of the macrophage itself or other nearby cells after release to the extracellular medium [9,10].

Monocyte and macrophage function has shown to be impaired under microgravity conditions for reviews see [4,11,12]. In microgravity substantial changes were detected in gene expression of monocytes and in gene induction associated with the differentiation of monocytes into macrophages [13]. When it comes to rapid-responsive molecular alterations in mammalian cells, short term microgravity provided by parabolic flight maneuvers is an ideal way to elucidate such initial and primary effects [14]. During a parabolic maneuver, an aircraft is “weightless” (residual acceleration in the range of 10^-3^ g, which could be better termed microgravity (μg) conditions) by flying on a Keplerian trajectory, described as an unpropelled body in ideally frictionless space subjected to a centrally symmetric gravitational field [15]. During this free-fall trajectory, the resultant of all forces acting on the aircraft other than gravity is nulled. However, parabolic flights using the European flight platform on board the Airbus A300 provide repetitive microgravity periods of 22 s only, interrupted by acceleration phases of 1.8 g and 1 g periods. Longer periods of microgravity are provided on satellites or the ISS (International Space Station). Ground-based facilities complement the gravitational research platforms. An established experimental approach is 2D clinorotation, enabling the rotation of a sample around one axis perpendicular to the gravitational field [16], thereby achieving the status of simulated microgravity or “functional weightlessness”. The condition of weightlessness is characterized by the lack of sedimentation and thus by a homogeneous distribution of particles. On the ground, this situation can be achieved by rotating a suspension of particles, which will still fall, but will be also forced on circular paths with decreasing radii through faster rotation of the system. The clinostat rotation has to be fast enough to achieve a situation where the rotated system no longer perceives the rapidly turning gravity vector (compensation of the gravitational force) and thus experiences “weightlessness” [17-21].

In this study we used NR8383 rat alveolar macrophages, which were considered to be good candidates to replace primary isolates because they display a similar response to stimulation in terms of superoxide production and changes in the concentration of intracellular calcium [22-24], and have proved to be a suitable experimental system [22]. In a combination of experiments using 2D clinorotation and real microgravity provided by several parabolic flight campaigns, we found that the oxidative burst reaction and phagocytosis in NR8383 macrophages depends on the gravitational force. We could demonstrate that the oxidative burst reacts rapidly and reversible to altered gravity conditions and therefore assume the oxidative burst, one of the key elements in the innate immune response and cellular signaling [25], to be strongly dependent on the gravitational force.

## Materials and methods

### Cell culture and assays

Cells of the cell line NR8383 (semiadherent, kindly provided by Karlsruhe Institute of Technology) were cultivated in Ham’s F12 medium supplemented with 10% fetal calf serum (both Biochrom) and 50 μM 2-mercaptoethanol (Gibco) and kept at 5% CO_2_ and 37°C. For most tests, cells were harvested and used immediately for the ground controls, clinostat and centrifuge experiments. For parabolic flights, no cell culture facilities could be provided on site. Therefore, cells were frozen in 1 ml freezing medium (RPMI medium with 20% fetal calf serum, 12% DMSO and 50 μM 2-mercaptoethanol) in several stocks of a defined cell concentration. These stocks were stored on dry ice, thawed in at least 20 ml of cold medium in the morning before each flight day and regenerated at ambient temperature for 30 min. After medium replacement (centrifugation for 8 min, 300 g), cells were adjusted to final concentration (see below) and incubated at 37°C up to 4 h before they were used for experiments. In single experiments, medium was supplemented with 0.3% methyl cellulose to prevent/delay cells from sedimentation.

### Luminol assay

Kinetic measurements using luminol (Sigma) were performed in the Synergy 2 reader (Bio Tek) after incubation in the pipette clinostat and the PMT clinostat. For measurements in microplates, cells were transferred directly after removal from the 1 ml clinostat pipettes. After adding 50 μl of a 10 mM luminol (Sigma) solution (diluted with borate buffer from a 100 mM stock in DMSO (Sigma) according to Pavelkova and Kubala [26]) to 170 μl cells (1*10^6^/ml) containing 3 U/ml horseradish peroxidase (Merck), the reaction was initiated with 70 μl of opsonified zymosan (Sigma) solution (prepared according to Allen [27] and monitored for 2 h). Measurements in the PMT-clinostat cuvette were performed with 560 μl cell suspension, 165 μl luminol solution, 33 μl horseradish peroxidase and 230 μl opsonized zymosan. Cell concentration was 7*10^5^/ ml. For mechanical controls, a cell-free, chemiluminescent solution was used: 165 μl lunimol (1 mM, including 2.5% NaOH), 230 μl H_2_O_2_ solution (0.3%) and 560 μl ammonium ferric citrate solution (saturated, than diluted 1:1000). After a high peak at the beginning, a stable signal was detected for 50 min.

### Nitro blue tetrazolium chloride (NBT)-assay

The NBT-assay was adapted on semiadherent cells [28]. NBT (Fluka) was coupled on opsonified zymosan [27] by incubating it in a 0.2% (w/v) NBT solution for 2 h at 37°C. Additionally, it was used in 0.2% (w/v) PBS solution to measure ROS-production in non-activated cells. 150 μl NBT-zymosan (or NBT-PBS) was mixed with 100 μl medium including 2.5*10^5^ cells. Incubation was at 37°C on the pipette clinostat and MuSIC for different periods and stopped by placing samples on ice. Cells were centrifuged at 2000 rpm for 2 min, after which pellets were fixed in 500 μl methanol (70%, Merck) and centrifuged. Cell pellets (including formazan from oxidized NBT) were lysed in 2 M KOH and the solution was mixed with 140 μl DMSO (Sigma). Absorption was measured at 630 nm in a microplate reader (Tecan Sunrise or GloMax Multi+, Promega).

### Phagocytosis assay

Phagocytosis was measured with FITC-labeled zymosan. Opsonized zymosan [27] was incubated with 0.4% (w/v) FITC (Fluka) for 30 min at 37°C in the dark. Afterwards, zymosan was washed up to 10 times, until the supernatant was no longer colored. Concentration was adjusted to the original, aliquots were stored at -20°C. 250 μl FITC-zymosan solution was mixed with medium containing 5*10^5^ cells. Incubation was at 37°C on the pipette clinostat for different periods and stopped by placing samples on ice. The cells were analyzed in a microplate reader (Spectra Fluor Plus, TECAN), kept on ice during the whole procedure. After transferring the cells into a microplate, 80 μl 0.4% trypanblue solution (Sigma) was added to quench the extracellular fluorescence. By centrifugation of the plate, cells were sedimented, and fluorescence of intracellular FITC-zymosan was measured from the bottom at 485 nm excitation and 535 nm emission. Control cells were kept on ice during incubation time, so that no phagocytosis took place. Relative fluorescent unit (RFU) values of controls were subtracted. To avoid artefacts due to different recovery of cell numbers of rotated and non-rotated cells (from the test tubes), RFU was calculated per 1*10^5^ cells after determination of recovered cell concentrations.

### 2D pipette clinostat

**Figure 1:**
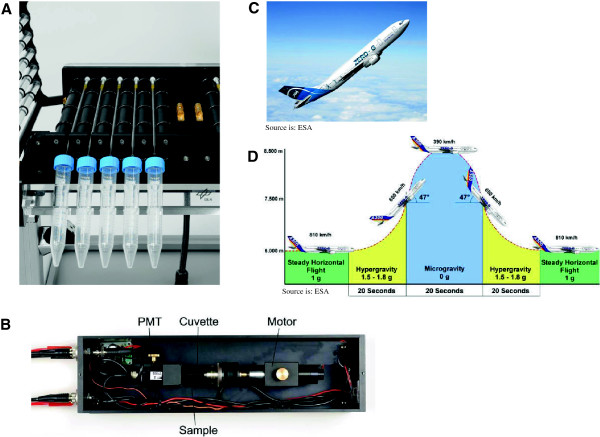
**Special equipment for experiments in simulated and in real microgravity.** Inside view of a pipette clinostat for parallel operation of up to 10 pipettes (Design J. Hauslage, DLR, Cologne) **(A)**, of the PMT clinostat with the photomultiplier, sample cuvette and motor **(B)**, parabolic flight aircraft Airbus A300B2-103 **(C)** and flight profile **(D)**.

A 2D clinostat (Figure 1A) adapted for the usage of pipettes (pipette clinostat) and the cultivation of mammalian cells (developed by Biomedical Science Support Center, DLR Cologne, Germany) were used for endpoint measurements of phagocytosis and oxidative burst (NBT). 1 ml pipettes (Falcon) had been filled with 500 μl – 1000 μl of cell suspension in a concentration of 1*10^6^ cells/ ml. Clinorotation at 60 rpm was performed at 37°C. Under the chosen experimental conditions (60 rpm, pipette diameter 3 mm) a maximal residual acceleration of 0.006 g is achieved at the border of the pipette. Clinostat speed was set at 60 rpm [21,29]. Up to 10 samples can be processed in parallel. After incubation, the suspensions were transferred immediately on ice to stop the reaction and for further processing. 1 g controls were treated the same way without rotation of the pipettes.

### Photomultiplier tube (PMT) clinostat

The PMT-clinostat [29] was used for luminol kinetic measurements during microgravity simulation (Figure 1B). It is equipped with one sample cuvette (diameter 4 mm, 3 cm length), which can be rotated at different speeds, (60 rpm in the present case, or without rotation for 1 g controls or parabolic flight experiments), and a PMT for the detection of light signals emitted by the samples. Under the chosen clinostat conditions the residual acceleration was less than ≤ 0.008 g. Cells (7*10^5^/ml) and reagents were prepared according to the luminol assay protocol, with measurement duration between 60 and 120 min. Data from the first 3100 s were analyzed. The clinostat was integrated in a heating box to provide 37°C independent of laboratory incubators.

### Centrifugeal systems

Two different centrifugeal systems at the Institute of Aerospace Medicine, DLR Cologne were used for hyper-*g* experiments at 1.8 g. The Multi-Sample Incubation Centrifuge (MuSIC) was applied for the analysis of phagocytosis. Cryotubes or Eppendorf cups (1.5 ml) were filled with the cell suspension and FITC-labeled zymosan, and then incubated at 37°C for 60 min. Further processing followed the description above. On the Short Arm Human Centrifuge (SAHC) luminol kinetics were performed with the static PMT clinostat. The clinostat tube was fixed on the platform perpendicular to the rotation axis of the centrifuge. Centrifugation was carried out for 45 min at 26.7 rpm, which results in 1.8 g at the position of the cuvette.

### Parabolic flight experiments

During the 13th DLR and the 51st ESA parabolic flight campaign, oxidative burst was studied online in real microgravity conditions using the PMT clinostat in a non-rotating mode and therefore as PMT sensor only (Figure 1B) in combination with the luminol assay. Single experiments during parabolic flights were also performed in the clinorotation mode to evaluate the quality of microgravity simulation. During each campaign, three flight days each with 31 parabolas were performed by an Airbus A300 (Figure 1C). The profile of one parabola is 22 s 1.8 g, 22 μg, 22 s 1.5 - 1.8 g (exact acceleration profiles are given in the corresponding figures, for an overview see Figure 1D). The in-flight experiment equipment consisted of a rack with a PMT clinostat and an interface to a laptop computer (pulse counter, power supply). The clinostat was inserted in an incubation box (37°C) with a small heater and a box with cooling packs for cooling of the syringes filled with activator fluids. A suspension of zymosan A in PBS (freshly opsonified with donor horse serum, 600 ± 200 particles/μl and stained with either FITC or Texas Red) containing 500 U/ml peroxidase (HRP) was prepared freshly and stored at -20°C. Luminol (100 mM in DMSO) was stored at -20°C. Zymosan and luminol were prefilled in syringes. In the morning of each flight day, 1 ml of the cell stock was thawed (see cell culture) and the cell suspension was filled into the two sample cuvettes for the two experiments of one flight day and stored at 37°C. Afterwards, prepared zymosan (including HRP) as well as luminol solutions were thawed. When the parabolic flight zone was reached, phagocytosis was started by injection of luminol and zymosan shortly before the first parabola in 1 g conditions. Two syringes filled with luminol and zymosan (sealed with caps) were connected to permanently installed parafilm-sealed cannulas in a septum at the sample cuvette and the zymosan and luminol were injected. Afterwards, the sample cuvette was installed in front of the photomultiplier (PMT), the experiment box was closed and the PMT started to record the luminescence data. During the 8 min break between parabola 15 and 16, the first sample cuvette was replaced by the second one and a new experiment was started by injection of a fresh set of luminol and zymosan. After the last parabola, PMT recording was stopped. Baseline experiments: All three flight days were simulated on the ground immediately after each flight with the same equipment and a new stock of cells and reagents in the Airbus A300 ZERO-G in the afternoon. Therefore, we can exclude potential influences of the hardware and environment in the Airbus.

### Statistics

At least four repeats were performed for each experiment. Statistical analysis was performed by calculating the arithmetic mean of the parallels with corresponding standard error (±SE; SE = SD/√n). All analyses were carried out by Origin 7.5 or PASW Statistics 18. Tests on normality were performed after Shapiro-Wilk, and in very few cases, outliers were determined by the Grubbs test. Significant differences were investigated by means of one way ANOVA with an ensuing Tukey HSD test. If two groups were compared, an unpaired Student t-test was performed. Significance levels are: α < 0.05 (*), α < 0.01 (**) und α < 0.001 (***). For a better graphical demonstration, all data were normalized by setting the means of non-exposed controls to the value 1. Afterwards, all single data were calculated as a ratio to control.

## Results

### ROS release strongly depends on gravity conditions

During the 13^th^ DLR and the 51^st^ ESA parabolic flight campaign, oxidative burst was studied in real microgravity conditions using a PMT clinostat and a luminol assay. In order to use identical hardware in all experimental set-ups, the PMT clinostat was used in the parabolic flights. The clinostat was either applied in the non-rotating mode, thereby measuring under the current accelerations (1 g, 1.8 g, microgravity), or in its operational mode (60 rpm). The latter configuration was used in periods of real microgravity to evaluate the quality of the simulation approach

**Figure 2:**
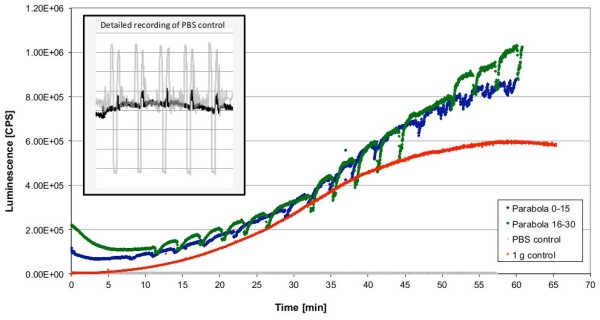
**Influence of parabolic flight on the ROS production visualized by luminol kinetics in the PMT clinostat (non-rotating) during the parabolic flights.** Cells were stimulated by opsonized zymosan as indicated. Luminescence was measured in counts per second (CPS). Two independent measurements during one flight day are shown in blue (stimulation before parabola 0) and green (before parabola 16). 1 g control experiment was performed on ground after each flight using the same experiment hardware, in flight-analogueconditions. Unstimulated control cells (PBS control) were not activated with zymosan.

**Figure 3:**
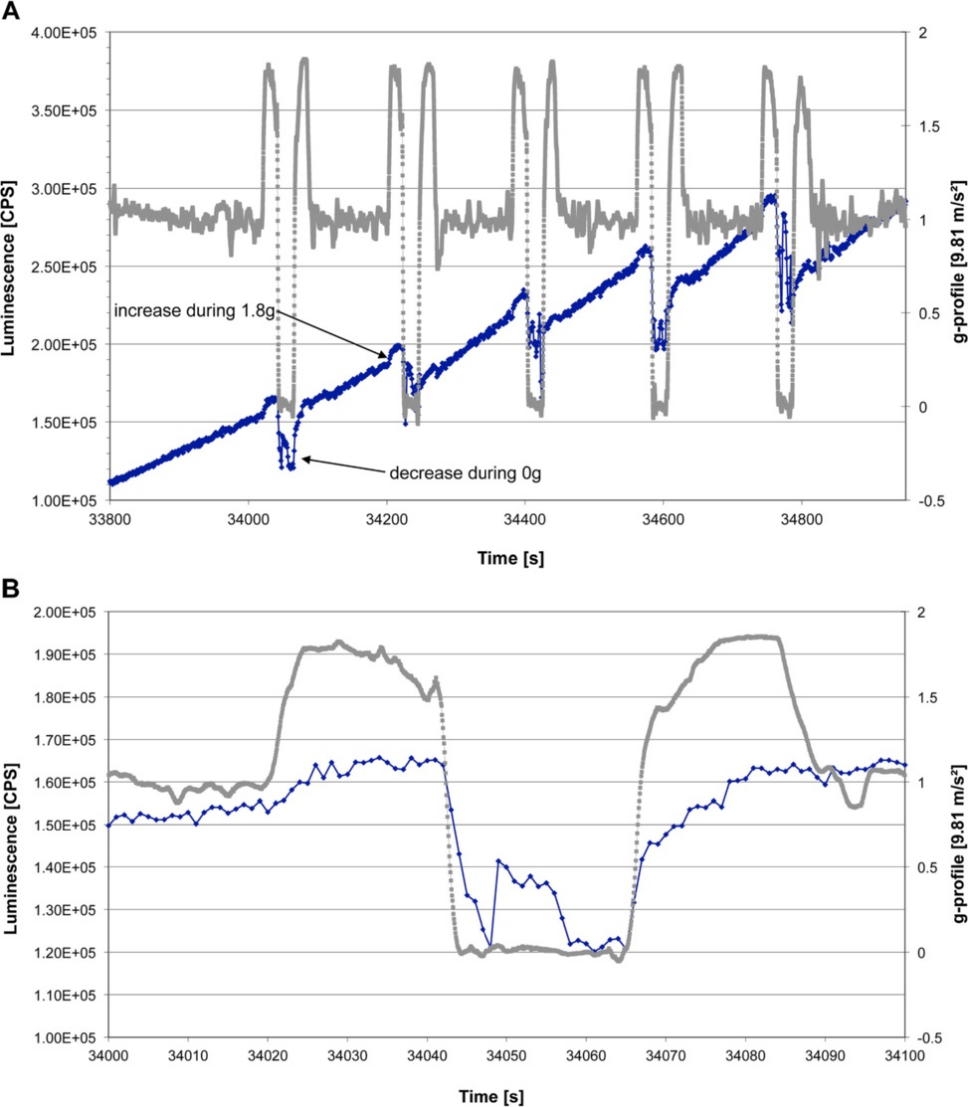
**Influence of changing g-forces during parabolic flight on the ROS production visualized by luminol kinetics in the PMT clinostat (non-rotating).** Cells were activated by opsonized zymosan stimulation before the first parabola. Luminescence was measured in counts per second (CPS). **A**. Detailed ROS production during parabolas 6–10 (blue) is shown in correspondence to g-profile (grey, kindly provided by Novespace). **B**. Detailed ROS production in response to altered gravity during parabola 6.

**Figure 4:**
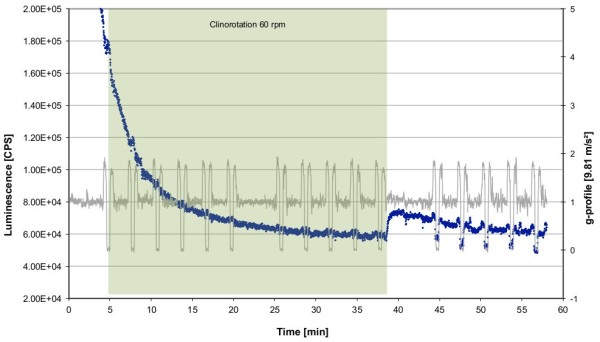
**Influence of changing g-forces during parabolic flight and simultaneous clinorotation on the ROS production visualized by luminol kinetics in the PMT clinostat.** Cells were activated by opsonized zymosan stimulation before the first parabola and clinorotated for 11 parabolas at 60 rpm. Luminescence was measured in counts per second (CPS). Detailed ROS production (blue) is shown in correspondence to g-profile (grey, kindly provided by Novespace). The clinorotated interval is grey shaded.

**Figure 5:**
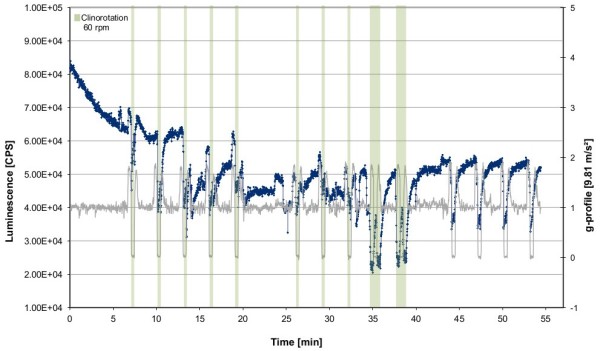
**Influence of changing g-forces during parabolic flight and clinorotation on the ROS production visualized by luminol kinetics in the PMT clinostat.** Cells were activated by opsonized zymosan stimulation before parabola 16 and clinorotated in μg at 60 rpm for 8 parabolas. In parabolas 24 and 25 clinorotation was performed during μg and hyper-g and rotation was then stopped. Luminescence was measured in counts per second (CPS). Detailed ROS production (blue) is shown in correspondence to g-profile (grey, kindly provided by Novespace). The clinorotated intervals are grey shaded.

**Figure 6:**
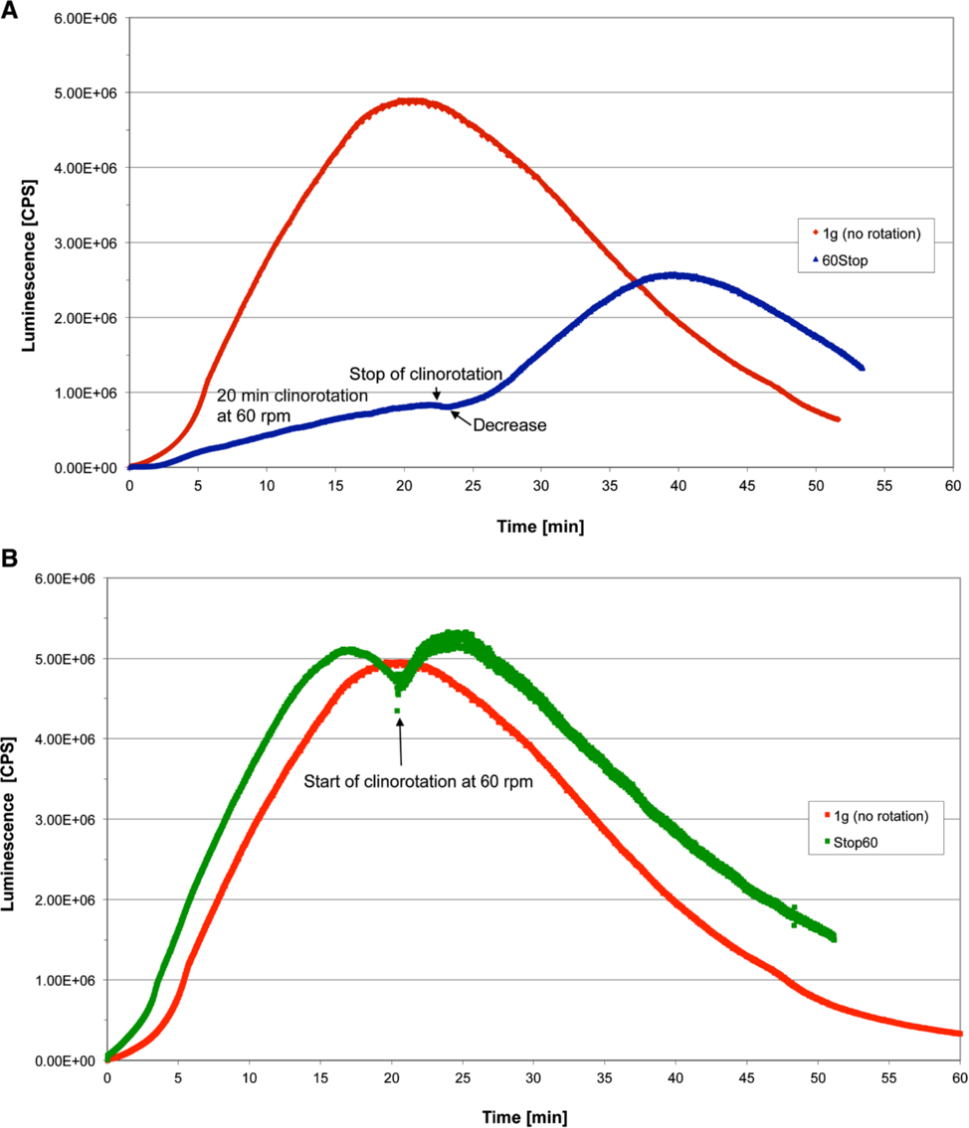
**Influence of two different clinorotation profiles on the ROS production visualized by luminol kinetics in the PMT clinostat.** Cells were activated by opsonized zymosan stimulation beforehand. Luminescence was measured in counts per second (CPS). **A** Profile 60Stop (60 rpm for 20 min, 1 g for 30 min, blue) in comparison to 1 g (no rotation) (red). **B** Profile Stop60 (1 g for 20 min, 60 rpm for 30 min, green) in comparison to 1 g (no rotation) (red).

**Figure 7:**
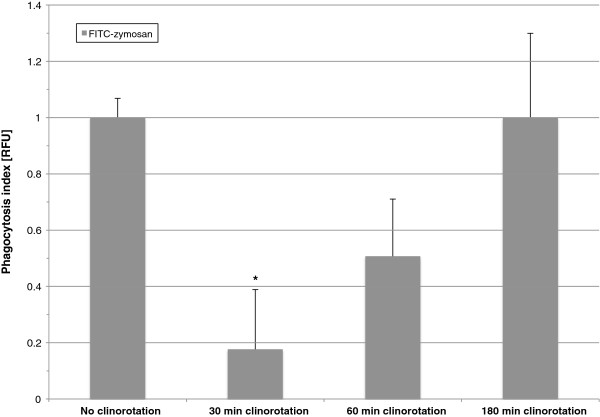
**Influence of clinorotation on the phagocytosis index determined by the incorporation of FITC-zymosan.** Cells were incubated with FITC-zymosan and directly subjected to rotation at 60 rpm for the indicated time periods. After rotation, phagocytosis was measured in relative fluorescent units (RFU) using a microplate reader. * p<0.05.

To investigate the generation of ROS under altered gravity conditions in the rat macrophage cell model, 12 measurements were performed during two parabolic flight campaigns with three flight days each (Figures 2, 3, 4, 5, and 6). Eight experiments (on four flight days) were carried out subjecting the cells to the accelerations occurring during parabolic flights (31 parabolas per flight day). Therefore, after activation with opsonized zymosan, four repeats could be performed for the first cell batch (activation before parabola 0, measurement until parabola 15) as well as for the second cell batch (activation before parabola 16, measurement until parabola 30). In the representative graphical presentation of the ROS generation (Figure 2), a generally increasing signal over time, caused by ROS-production after zymosan-ingestion, could be recorded. Compared to the luminol kinetics of freshly harvested cells (Figure 6A, 1 g, peak after 20 min), burst curves during parabolic flights appeared lower and slightly delayed, only attaining a more prominent profile with an increasing number of parabolas. This observation can probably be attributed to the gradual functional relatively short recovery phase of the cells after thawing, since the 1 g ground control cells exhibited similar kinetics (see Figure 2). Oxidative burst kinetics after stimulation with opsonized zymosan showed an increase in ROS production within the first 20 min, after which the luminol signal dropped after reaching a peak (Figure 7, 1 g).

Of particular interest is how repeated signal drops were monitored that correlated perfectly with the interval of the μg-phases of the parabolas (compare to Figure 3 with corresponding gravity profile). No relevant differences in the principal kinetics between the first and second cell batch could be noticed, except for a higher start-signal of the second cell batch (parabolas 16 – 30), which already experienced different gravity conditions in a non-activated state. This initially higher ROS signal drops within the first minutes to a basal level before the actual zymosan-digestion starts. The circumstantial variance of the overall signal strength is assumed to be due to the varying regeneration periods of the cells after thawing. A correlation of the ROS production with the parabolic flight profile (Figure 3) revealed recurring increases of the signal during each initial hypergravity phase. ROS-production of cells, that were not activated with zymosan (Figure 2, PBS control) remained at a basal level with short signal increases at the end of the μg phases and during the second hyper-g phases (Figure 2, grey box). However, no signal drops could be observed.

On two other flight days (four experiments), clinorotation was performed during the parabolas to investigate the quality of microgravity simulation provided by the PMT clinostat. In an initial approach, cells were rotated permanently at 60 rpm during the first 11 parabolas. Clinorotation was then stopped, and the cells were subjected to the normal parabolic flight profile for 5 parabolas (Figure 4, clinorotation interval is grey-shaded). Onset of the rotation reduced the ROS production immediately and almost entirely masked the effect of different gravity conditions during the parabolas. During the following five parabolas without clinorotation, the pattern of signal gain and drop recovered. However, the relative amplitude of the signal seemed reduced and there was no gradual increase after zymosan activation compared to earlier recordings. In a second approach, the cells were rotated at 60 rpm only during microgravity phases for eight parabolas (Figure 5, clinorotation intervals are grey-shaded). Upon onset of rotation, there was a drop in ROS production, which indicated that clinorotation did not interfere with the real microgravity conditions during the parabolic maneuver. During hypergravity conditions without clinorotation, an earlier signal increase could be observed. In parabola 9 and 10, clinorotation was performed during hypergravity and microgravity, resulting in a pronounced signal drop during the microgravity phase. During the following five parabolas without any rotation, the previously described signal pattern (see Figure 3) was restored. This demonstrates effective simulation of microgravity by the PMT clinostat, comparable to microgravity conditions generated by a parabolic flight maneuver.

### Initial activation of the burst reaction is highly sensitive to altered gravity

Different acceleration profiles were tested after zymosan-induced oxidative burst. In the first profile, clinorotation was performed for 20 min at 60 rpm and measurement continued at 1 g for 30 min (“60Stop”). In a second profile, the cells were kept at 1 g for 20 min and then rotation started at 60 rpm for 30 min (“Stop60”). Figure 6 shows the ROS kinetics of both profiles. In the “60Stop” profile (Figure 6A), the oxidative burst was initially suppressed during clinorotation. Stop of the clinostat resulted after a short signal decrease in a rapid ROS production. However, the slope was not as steep as in 1 g and the maximum peak not as high. In the case of “Stop60” (Figure 6B), the macrophage cell line demonstrated normal ROS production at 1 g, but start of rotation induced a rapid signal decrease for a few seconds followed by recovery. We assume that signal recovery resembles more a re-activation than a continuation of the oxidative burst reaction, as an additional peak value is generated. The kinetics are similar to cells in 1 g conditions, apart from the interruption by the onset of clinorotation (Figure 6B). Table 1 shows the statistical analysis of the different profiles. The “60Stop” profile led to highly significant alterations in AUC, maximum peak height and the time until the peak is reached. Therefore, exposure to clinorotation during the activation phase resulted in a persistent repression of oxidative burst. In contrast, re-activation of oxidative burst occurred during clinorotation if the cells were previously activated under 1 g conditions. We therefore suppose that the gravity-sensitive steps are located in the initial activation of the burst reaction.

**Table 1:** Influence of three different clinorotation profiles in comparison to 1 g and 1.8 g on the ROS production determined by luminol kinetics

		**AUC**	**Maximum**	**Time to maximum**
1 g	Mean	8750669848	4946100	1462
	±SE	1151580523	101211	255
	n	15	15	15
60stop	Mean	3784575841***	2476726***	2377***
	±SE	389686291	411176	188
	n	5	5	5
Stop60	Mean	9807343407	5211433	1382
	±SE	492287332	419108	121
	n	5	5	5
PF profile	Mean	9241240447	4826909	1413
	±SE	935625878	518891	53
	n	5	5	5
1.8 g	Mean	18867748089***	11732458***	2338***
	±SE	2654166856	442477	252
	n	5	5	5

Cells were activated by opsonized zymosan stimulation beforehand.Luminescence was measured in counts per second (CPS) and quantified by calculating the area under the curves (AUC, total ROS production), the maximum peak height (Maximum) and the time to the maximum peak hight (Time to maximum). Values of normalized data are listed as means ± standard error (SE). n, number of replicates; 60Stop, 60 rpm for 20 min followed by 1 g for 30 min; Stop60, 1 g for 20 min followed by 60 rpm for 30 min; PF profile, simulation of gravitational changes during a parabolic flight; ***p < 0.001.

### Delayed phagocytosis and reduced phagocytosis-induced oxidative burst during clinorotation

**Figure 8:**
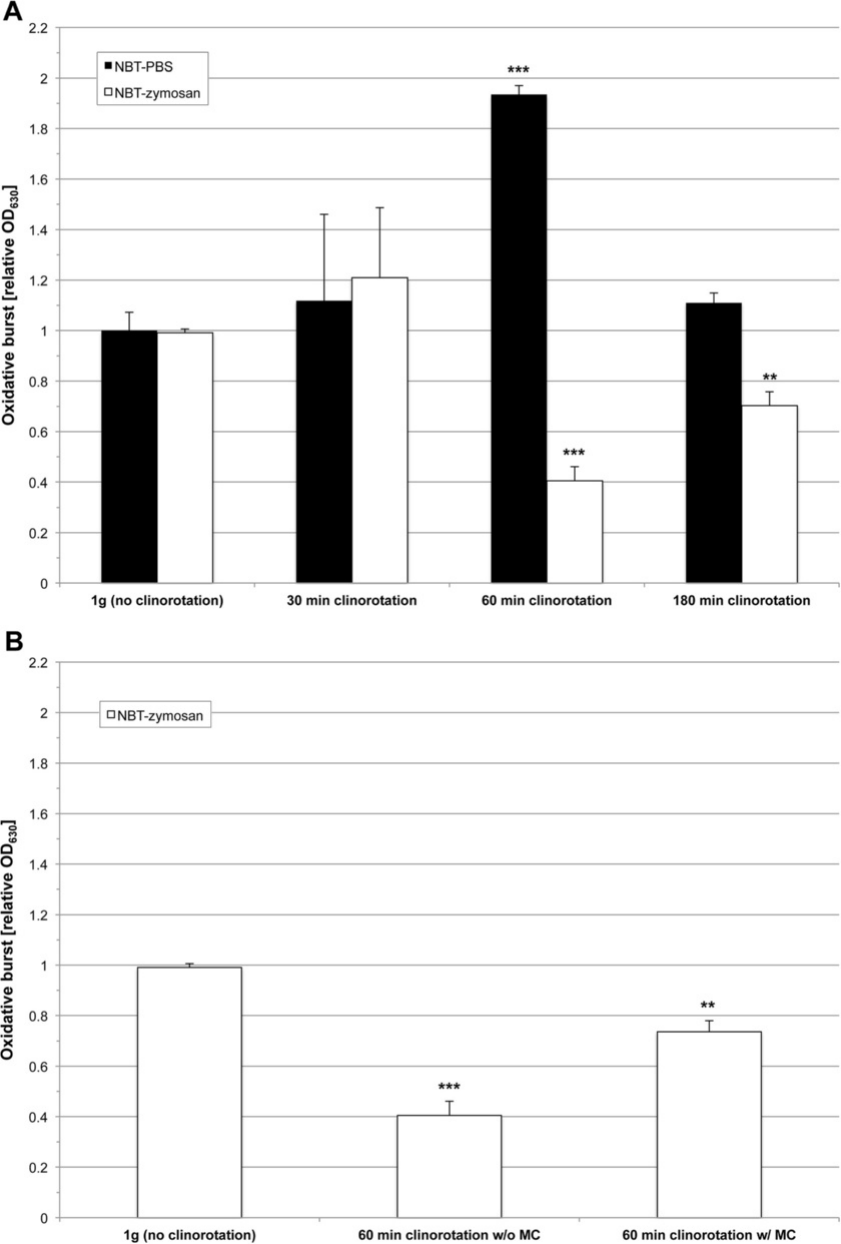
**ROS production during clinorotation, phagocytosis and sedimentation. A**. Influence of clinorotation on the phagocytosis-mediated and phagocytosis-independent ROS production determined by the NBT assay. Cells were incubated with NBT-zymosan (white bars) or NBT-PBS (black bars) and directly subjected to rotation at 60 rpm for the indicated time periods. After rotation, ROS production was measured in relative optical density (OD) at 630 nm using a microplate reader. **B**. Influence of clinorotation and sedimentation on the phagocytosis-mediated ROS production determined by the NBT assay. Cells were incubated with NBT-zymosan (white bars) with or without methyl cellulose (MC) and directly subjected to rotation at 60 rpm for 60 min. Sedimentation effects in control cells were minimized using a supplementation of 0.3% MC. After rotation, ROS production was measured in relative optical density (OD) at 630 nm using a microplate reader. **p<0.01; ***p<0.001.

To determine the oxidative burst reaction of activated versus non-activated cells under simulated microgravity conditions, we performed ROS measurement by using the Nitro blue tetrazolium (NBT) assay on zymosan-activated as well as untreated cells. Furthermore, phagocytosis was determined by the ingestion of FITC-labeled zymosan. For the latter, the cells were mixed with FITC-labeled zymosan and subjected to clinorotation for 30, 60 and 180 min. As these experiments were carried out in an old type of pipette clinostat, where only two replicates could be performed at the same time, data were normalized by setting the means of the controls of one experiment run to 1. Phagocytosis was only significantly reduced at the first endpoint measurement after 30 min of clinorotation (Figure 7). The fact that the other time points revealed no difference to 1 g indicates that phagocytosis was delayed but still functioning during clinorotation. The production of ROS measured by NBT changed differently in activated (phagocytotic) compared to non-activated cells. Phagocytosis-mediated ROS production was reduced by clinorotation after 60 min, whereas phagocytosis-independent ROS production was enhanced after 60 min (Figure 8A). Minimization of sedimentation as an inhibiting effect on phagocytosis-dependent oxidative burst was also detectable in control experiments by the application of 0.3% methyl cellulose to the medium (Figure 8B). Viability assays with trypan blue staining were carried out with aliquots of each sample after each experiment. There was no significant influence of clinorotation on the viability of the cells (data not shown).

### Long-term preconditioning in simulated microgravity enhanced ROS release

**Figure 9:**
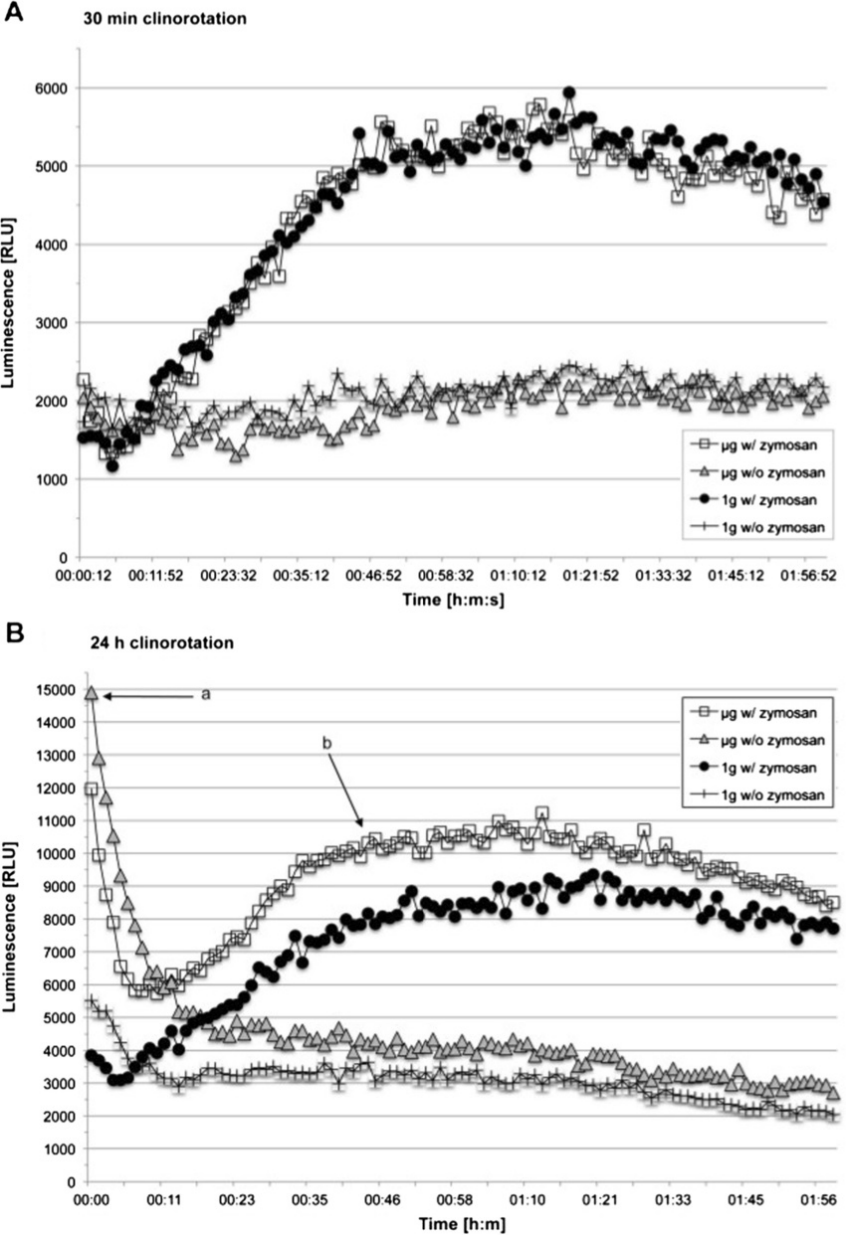
**Influence of previous clinorotation in comparison to permanent 1 g exposure on the ROS production visualized by luminol kinetics in a microplate reader.** Cells were activated by opsonized zymosan directly after clinorotation (w/zymosan) or left untreated (w/o zymosan). Luminescence was measured in relative luminescence units (RLU) per minute. **A**. Cells were clinorotated for 30 min previous to measurement. **B**. Cells were clinorotated for 24 h previous to measurement.

Since we found that ROS release was inhibited in microgravity (Figures 2, 3, 6), we tested whether short-term (30 min) or long-term (24 h) preconditioning in simulated microgravity affected ROS release in 1 g (Figure 9). Therefore, phagocytosis-dependent and –independent oxidative burst was measured in 1 g after incubation of the cells in the pipette clinostat for 30 min as well as for 24 h, respectively. Baseline ROS production of resting cells (without/before zymosan activation) was investigated comparing clinorotated samples to 1 g controls. After 30 min of clinorotation, no significant difference between the phagocytosis-dependent and phagocytosis–independent oxidative burst reaction could be detected compared to the respective 1 g control (Figure 9A). However, after 24 h of clinorotation, the amount of ROS production upon zymosan stimulation was significantly higher compared to the 1 g control (Figure 9B). Additionally, a high initial ROS-signal before start of phagocytosis was observed, which dropped to an almost basal level within the first 10 minutes, before the actual ROS-production upon zymosan stimulation starts. Therefore, oxidative burst is represented by the relative activation index, which is calculated from the quotient of the initial relative luminescence unit (RLU) and the maximum RLU peak. The letters “a” and “b” in Figure 9B indicate the phases which were used for calculation of activation indices. All given values are relative output data of a photomultiplier tube. The differences in signal strength between the 30 min and 24 h values can be attributed to inter-experimental variations rather than to an overall increase in oxidative burst. Table 2 presents the relative activation (baseline activation at point “a” compared to zymosan activation “b”) for all tested incubation times. Fluctuations of the values for 1 g controls were low, which indicated a low ROS production at point “a” before zymosan stimulation. In contrast, clinorotation resulted in higher baseline ROS production, which increased after longer incubation times. After 24 h of clinorotation, baseline ROS production was 20% higher than the phagocytosis-induced oxidative burst “b”. This initial effect of long-term clinorotation seemed to disappear after about 15 min in 1 g, which indicated that the cells are very quickly capable of re-adapting to 1 g conditions. Nevertheless, zymosan-activated cells, which experienced long-term clinorotation (24 h), showed an increased oxidative burst reaction even after 1 h at 1 g (see Figure 9B).

**Table 2:** Ratio of activation by clinorotation compared to zymosan activation after increasing incubation times in the clinostat

**Relative activation**			
		**Clinorotation**	**1 g**
	**n**	**Mean ± SD**	**Mean ± SD**
10 min	12	0.01 (±0.1)***	-0.18 (±0.12)
30 min	12	0.42 (±0.13)**	0.29 (±0.09)
1 h	12	0.25 (±0.07)***	0.006 (±0.07)
3 h	12	0.66 (±0.25)***	-0.02 (±0.07)
24 h	12	1.2 (±0.38)***	0.45 (±0.16)

Quotients are given as means of four independent experiments with three replicates having standard deviation (mean ± SD).

### Hyper-g increases the oxidative burst reaction of zymosan-activated cells

**Figure 10:**
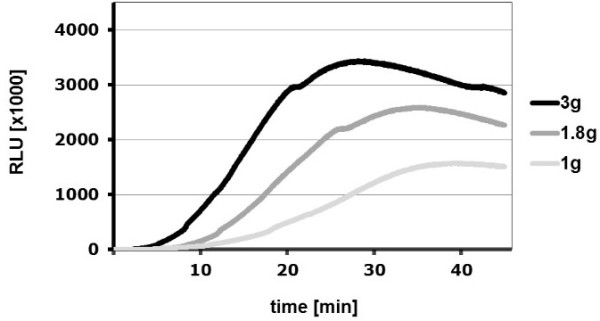
**Influence of centrifugation on the Short Arm Human Centrifuge (SAHC) on the ROS production visualized by luminol kinetics in the PMT clinostat.** Cells were activated by opsonized zymosan stimulation beforehand and subjected to 1.8 g, 3 g or to 1 g generated by the PMT clinostat within the SAHC. Luminescence was measured in relative light units (RLU). Data reflects one representative experiment of at least three replicates.

**Figure 11:**
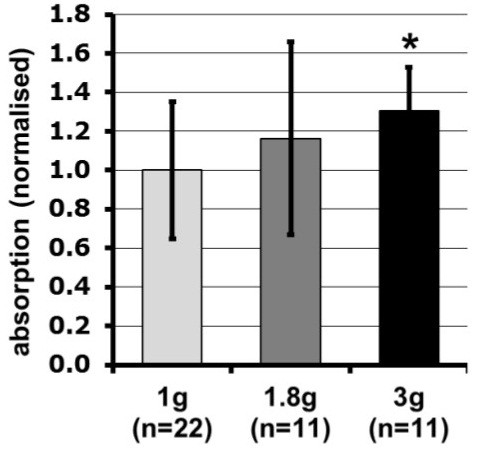
**Influence of centrifugation on the Multi Sample Incubator Centrifuge on the phagocytosis-mediated superoxide production determined by the NBT assay.** Cells were incubated with NBT-zymosan and directly subjected to rotation at 1.8 g and 3 g for 45 min. After centrifugation, ROS production was measured in relative optical density (OD) at 630 nm using a microplate reader (Promega). *p<0.05.

Because the oxidative burst signal increased during the hypergravity phases of parabolic flights, we performed luminol kinetic measurements on the Short Arm Human Centrifuge (SAHC, DLR, Cologne) to verify this finding using the identical hardware (PMT clinostat, non-rotating). ROS production in hypergravity (1.8 g and 3 g, Figure 10) was indeed increased and peak ROS release in 3 g and 1.8 g was higher, occurring earlier than in 1 g. Importantly, ROS release was enhanced with increasing gravitational force (3 g < 1.8 g < 1 g). However, the production of superoxide, measured by the reduction of NBT during exposure to hypergravity (MuSIC), was not significantly increased at 1.8 g, but only at 3 g (Figure 11). Since NBT-zymosan signals were not altered during 1.8 g centrifugation, increased ROS production in 1.8 g was probably not the consequence of altered phagocytosis. However, centrifugation in 3 g increased NBT-signals, which indicated an enhanced phagocytosis-mediated oxidative burst.

### Rapid and reversible reduction of ROS release in simulated weightlessness

**Figure 12:**
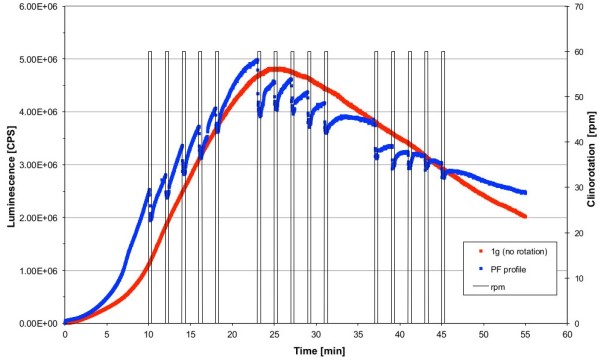
**Simulation of μg-phases of a parabolic flight by a clinorotation profile and influence on the ROS production visualized by luminol kinetics in the PMT clinostat.** Cells were activated by opsonized zymosan stimulation beforehand. Luminescence was measured in counts per second (CPS). Blue, ROS production by cells subjected to the parabolic flight simulation (PF profile); red, ROS production by cells subjected to 1 g (no rotation profile); grey, rotation profile in rounds per minute (rpm).

Finally, we used clinorotation to simulate the short and repeated microgravity phases during a parabolic flight. Corresponding to the parabolic flight profile, each parabola was simulated by rotation at 60 rpm for 22 s followed by a break of 98 s. And each set of 5 parabolas was separated from the following set by a 5 min break. The following clinostat profile was performed to simulate a parabolic flight: (I) rotation for 22 s at 10, 12, 14, 16, 18 min; (II) break of 5 min; (III) rotation for 22 s at 23, 25, 27, 29, 31 min; (IV) break of 5 min; (V) rotation for 22 s at 37, 39, 41, 45 min; (VI) stop of measurement after 50 min. During the simulated parabolic flight profile, we detected a decrease in ROS production at the onset of rotation (Figure 12), closely resembling the parabolic flight kinetics (Figure 2, 3). After the simulation stop, the ROS release immediately followed the 1 g kinetics. Therefore, we could confirm our findings during the real microgravity periods provided by parabolic flights in simulated microgravity by using 2D clinorotation at 60 rpm. Slight differences in the initial burst intensity between the 1 g and the rotated treatment were due to normal variability of different cell batches.

### Control experiments

Several control experiments (at least three independent repeats) were performed for the different experimental setups to avoid misinterpretation of the results. To exclude mechanical interference factors caused by vibrations, chemical luminescence was measured without cells in the PMT clinostat. During parabolic flights as well as during centrifugation on the SAHC, vibrations are relatively strong. Therefore, we performed further tests on a vibration platform (Vibraplex, DLR Cologne) simulating the vibrations with frequencies ranging from 0.2 Hz to 14 kHz induced by the engines of an airplane [30]. No changes in the luminol signal strength could be assigned to those mechanical interference sources. Even strong shaking and throwing did not alter the signals. If activated cells were shaken, their oxidative burst signal increased over the shaking time, but the signals never declined (as is the case for microgravity or during clinorotation). To exclude the possibility of signal reduction by quenching effects caused by floating cells, dead non-activated cells were clinorotated in chemiluminescent solution (generated by a commercial glow stick, Eurolite, Behr Angelsport, Ladenburg, diluted 1:98 with PBS). Luminescence was measured at 60 rpm and 1 g. Shortly before and after measurement, morphology and viability of the cells was judged using trypan blue staining. The cells appeared dead by the inclusion of trypan blue, yet morphologically intact. Onset of the rotation generated a quick increase of the signal caused by the mixing of the solution, although the signal returned immediately to the initial level. No reduction in signal strength caused by rotation could be detected. Therefore, oxidative burst inhibition observed under real and simulated microgravity is not an artifact caused by quenching effects.

## Discussion

Macrophages play a major role in the body’s defense against bacteria, viruses, and foreign particles in the lungs and tissue. This is achieved in part by the oxidative burst, a physiological response to soluble and particulate agonists consisting of the production of superoxide by the nicotinamide adenine dinucleotide phosphate (NADPH) oxidase [31]. According to the classic model of activation of the NADPH oxidase, receptor-mediated release of diacylglycerol and inositol trisphosphate by phospholipase C causes activation of protein kinase C. This leads to phosphorylation of cytosolic components of the NADPH oxidase, in particular p47^PHOX^, and assembly of the functional enzyme in the plasma membrane. Phospholipase D (PLD) is also activated during the respiratory burst in neutrophils and is necessary for superoxide production with many stimuli in those cells [32-34]. Superoxide generated by the NADPH oxidase is rapidly converted to H_2_O_2_ by spontaneous and enzymatic dismutation. A microbicidal function, usually in conjunction with phagocytosis, is attributed to these reactive oxygen species.

Indeed, immune system dysregulation has been demonstrated to occur during and immediately following spaceflight [35]. In a study including four space shuttle missions and 25 astronauts, the astronauts’ monocytes exhibited reductions in their ability to engulf *Escherichia coli*, elicit an oxidative burst, and degranulate following 5–11 days of spaceflight. The phagocytic index was significantly reduced, accompanied by changes in the expression of CD32 and CD64 [36,37]. In nine short-duration Space Shuttle crewmembers, constitutive monocyte expression of CD62L was reduced following spaceflight, which indicates a functional disability of monocytes in adhesion and tissue migration [35]. Following LPS stimulation of monocytes, postflight expression of IL-6, TNFalpha, and IL-10 were significantly reduced [35]. Immunological alterations during spaceflight could of course be the consequence of stress-reactions of the whole organism as well as of direct microgravity effects at the cellular level. In a study using gravitational changes during parabolic flights as a stress model, an increased leucocyte number with a significant elevation of the PMN fraction was detected [38]. The spontaneous hydrogen peroxide production by PMNs did not change and the capability of PMNs to produce H_2_O_2_ in response to soluble stimuli such as fMLP and TNF-alpha, calcium ionophore (A23187), and phorbol myristate acetate (PMA) was increased [38].

At the cellular level, a previous study reported that oxidative burst activity was significantly decreased in nonadherent promyelocytic (HL-60) cells in simulated microgravity conditions generated by rotating wall vessels (RWVs) [39]. Alterations of the cytokine secretion profile and, in particular, of inflammatory chemokines, as well as a decrease of the proteasome activity, were observed in response to RWV exposure in human myelomonocytic U937 cells [40].

In our experiments we could demonstrate in real microgravity (parabolic flights, Figures 2, 3, 4, and 5), in simulated microgravity (2D clinostat, Figures 6, 7, 8, 12) and in hypergravity (centrifuges and parabolic flight, Figures 2, 3, 4, 5, 10, 11) that the ROS release during the oxidative burst reaction upon zymosan stimulation depends significantly on gravity conditions. ROS release 1.) is reduced in microgravity, 2.) is enhanced in hypergravity and 3.) responds rapidly and reversible to altered gravity within seconds. Experiments in real microgravity were also conducted with different cell batches and during different sequences of parabolas (Figures 2). Since the general kinetics after zymosan stimulation was comparable, we were able to confirm the first experiment set and demonstrated also a robust and reproducible effect, not only between the several parabola, but also between different cell batches and after previous exposure to sequenes of altered gravity conditions. Furthermore, the state of activation of the macrophages is important for the ROS-response to microgravity. This was demonstrated in different approaches. Figures 2, 8A and 9B show that non activated cells, previously or currently exposed to microgravity react with increased ROS-production.

The cells used for parabolic flights had to be restored after freezing every morning before flight due to the lack of cell culture facilities on site. Therefore, the luminol curves did not show a “normal” kinetic (quick increase and peak after about 20 min with following decrease like in Figure 6A), but was delayed in their response to zymosan stimulation (Figure 2). This is caused by the short recovery time of the cells, but very likely also by a reduction of phagocytosis-initiation, like it was also demonstrated during clinorotation (Figure 7). Nevertheless, the response to zymosan during altered gravity conditions differs strongly to the non-activated control cells indicating that phagocytosis-mediated oxidative burst was measured during parabolic flight, even during clinorotation. The fact that there was no zymosan-mediated signal increase in cells exposed to parabolic flight and clinorotation, could be explained by reduced phagocytosis in microgravity. However, we assume that the cells were in an activated state, as they show drops in ROS release (after clinorotation stops and during clinorotation in μg), a cellular response not exhibited by resting cells.

The very fast responses of the oxidative burst during parabolic flight further indicate, that a direct effect on the signalling pathway can be expected rather than alterations of the phagocytosis.Our results could provide an explanation of the reduced phagocytosis and oxidative burst reaction in monocytes from astronauts after spaceflight [36,37] as a direct effect of microgravity on cells of the monocyte-macrophage system. This is in accordance with the previously reported significantly decreased burst reaction in cultured promyelocytic HL-60 cells in simulated microgravity [39]. Although we found very rapid and reversible *in vitro* effects of altered gravity on the oxidative burst reaction, pre-conditioning effects were also detected in simulated microgravity (Figure 9). Thus, the effect of microgravity on the oxidative burst reaction in the monocyte-macrophage-system *in vivo* clearly requires further investigations.

In clinorotation experiments, human monocytic cells responded with tyrosine-phosphorylation of several proteins, whereas in PMA-stimulated monocytic cells, tyrosine-phosphorylation was nearly abrogated [41]. These observations are supported by phosphorylation of c-jun and the binding of phospho histone H3 to c-jun by clinorotation [41]. Such effects would inhibit the specific and directed action of antigen-presenting cells and phagocytes, but would stimulate large numbers of phagocytes to execute non-specific and non-directed phagocytosis, such as in bone tissue. Thus, it could be possible that microgravity puts resting macrophageal cells into a state of alert and into a state of non-specific activation, whereas activated macrophageal cells are inhibited, as demonstrated after long-term clinorotation for NR8383 (see Figure 9).

2D clinostats complement the gravitational research platforms by a solely-ground-based device, which enable the rotation of a sample around one axis perpendicular to the gravitational field [15]. The clinostat rotation has to be fast enough to achieve a situation where the rotated system no longer perceives the rapidly turning gravity vector (compensation of the gravitational force) and thus experiences “weightlessness” [17,20]. In our study we found an impressive accordance between the experiments in simulated and in real microgravity (Figures 2, 3, 4, 5, 6, 7 and 8, 12), supporting the establishment of clinostat experiments as a valuable tool to simulate microgravity for suspension cell cultures [21]. We performed control experiments to exclude a direct effect of altered gravity conditions on the measurement system and therefore artificially generated signal alterations. The μg-induced signal drops in zymosan-stimulated cells did not occur in the μg-phases during permanent clinorotation, and also not in non-activated cells. In the case of a direct impact of altered gravity on the PMT, the signal drops and peaks would also have appeared during clinorotation, as only the cuvettes were rotated but not the PMT. Therefore, the decrease of the signal cannot be a technical measurement artefact in microgravity. In summary, the described effects were not only substantiated by two independent parabolic flight campaigns but also in 2D clinorotation experiments and thus in simulated microgravity on ground. We not only demonstrated the gravi-sensitivity of oxidative burst reaction in well controlled ground-based experiments, but also in real microgravity conditions, an experiment combination which was described as the “ultimate validation” in a recently published review [21].

Zymosan, a glucan with repeating glucose units connected by β-1,3-glycosidic linkages, induces inflammatory signals in macrophages through toll-like receptors TLR2 and TLR6 and has served as a model for recognition of microbes by the innate immune system for over 50 years [42]. Thus, gravitational forces may act in the pathways between the receptors and the NADPH oxidase, but the very rapid and reversible effect of gravitational forces on ROS release (Figure 2) suggests a direct and rapid effect on the ROS releasing enzyme, whereas the requirement of gravitational forces during application of zymosan for a full oxidative response (Figure 6) suggests an effect within early events of the signaling pathway.

The leukocyte NADPH oxidase belongs to a group of plasma membrane-associated enzymes found in professional phagocytes and B lymphocytes. By using NADPH as an electron donor, it catalyzes the production of superoxide (O_2_^-^). The O_2_^-^ then serves for the downstream production of several reactive oxidants, including oxidized halogens, free radicals, and singlet oxygen to kill invading microorganisms. The NADPH oxidase comprises five components: cytosolic p40^*PHOX*^ (phagocyte *ox*idase), p47^*PHOX*^, p67^*PHOX*^, and secretory vesicle membrane located p22^*PHOX*^ and gp91^*PHOX*^ also known as cytochrome b_558_. Upon exposure of the resting cell to adequate stimuli, the cytosolic complex migrates to the membrane, where it assembles the active oxidase [43]. During phagocytosis, the plasma membrane is internalized and the outer membrane surface reverted to face the interior of the formed vesicle. Then, the NADPH oxidase discharges O_2_^-^ into the vesicle, exposing its lumen to a deadly mixture of corrosive agents [44,45]. The intracellular activation of a phagocyte is initialized by the phosphorylation of p47^*PHOX*^ and the subsequent translocation of the entire cytosolic complex to the membrane, where it associates with cytochrome b_558_ and forms the active oxidase [43,46,47]. The phosphorylation of p47^*PHOX*^ is regulated by protein kinase A [48]. Inhibition of MAP kinase, an upstream effector of ERK, and of p38 can prevent oxidase activation [49,50] suggesting that both ERK and p38 participate in the activation of NADPH oxidase. MAP kinases have been previously suggested as molecules for cellular signal transduction of altered gravitational forces [5,41]. Furthermore, activation requires the participation of two guanine nucleotide-binding proteins (G proteins): cytoplasmic Rac2 and membrane located Rap1A. Rac2 belongs to the Rho family of G proteins, known principally for their function in cytoskeleton regulation. Rap1A is a member the Ras family regulating cell proliferation. During activation, Rac2 binds guanosine triphosphate (GTP) and accompanies the cytosolic complex to the membrane, while cytochrome b_558_ and Rap1A are delivered to the cell surface by fusion of the secretory vesicles with the plasma membrane [51-53].

Activation of NADPH oxidase also includes the process of surface adherence in neutrophils involving integrins and association with considerable changes in the cytoskeleton and the production of O_2_^-^ compared with suspended cells. It was found that upon activation of neutrophils, all the O_2_^-^ producing activity and portions of the oxidase components are found in the cortical cytoskeleton [54,55]. Studies suggest that these components are translocated from the membrane surface to the cytoskeleton together with β_2_ integrin receptors upon activation by binding of antibodies and complement factors. Along the way, O2^-^ is produced [56,57]. In neutrophils deficient in CD18, a common subunit of β_2_ integrins, another pathway is used for activation of NADPH oxidase. There, the leukocyte response integrin (LRI) recognizes the basement membrane protein entactin and acts in association with the integrin-associated protein (IAP), thus activating the production of reactive oxygen species [58]. Furthermore, direct associations between particular cytoskeletal proteins and the NADPH oxidase have also been described. For instance, the human analog of coronin, a protein involved in motility of *Dictyostelium,* associates with p40^*PHOX*^ and accumulates around phagocytic vesicles [59]. These findings suggest a major role for the intact cytoskeleton in NADPH oxidase activation and defense against invading microorganisms.

Several studies demonstrated modifications of the cytoskeleton in microgravity [60-62]. Even a few minutes of microgravity affected the cytoskeleton of lymphocytes, astrocytes, neurons and glial cells, disorganizing microtubules, intermediate filaments and microfilaments [63-65], whereas changes during 22 seconds were reported in the F-actin and cytokeratin cytoskeleton in follicular thyroid cancer cells [66]. Paradigms of cellular mechanical force sensing have been reviewed by Orr et al. [67]. According to the tensegrity model [68], the whole cell is a pre-stressed structure [69,70], with tensions generated by the actin-myosin network. The folding state of cytoskeletal associated proteins, which creates or masks binding sites for other proteins, depends on the strains in the actin network reviewed in Vogel and Sheetz, [71]. Forces to the actin network could be therefore transduced in altered binding of signal proteins to the cytoskeleton. Consequently, microgravity may reduce the force inside the actin network, which could be then transduced into a certain biochemical signal by cytoskeleton-associated proteins. Interestingly, Rho kinase has been found to regulate the intracellular micromechanical response of adherent cells [72], and small G proteins are discussed as having a significant role in mechanotransduction [73]. Both protein families are important parts of the NADPH oxidase activation pathways [51-53].

Our results indicate that 1 g conditions are required for full activation during 1.) an initial activation step (see Figure 7A and 7B) and during 2) the release of ROS (see Figures 2, 3, 4 and 5). Since ROS release is reduced and restored very rapidly and reversible within seconds upon altered gravity, it assumes a direct effect at the level of the NADPH oxidase complex, which has been reported to be closely associated with cytoskeletal dynamics [54,55]. Additional potential regulators could include MAP kinases, rho kinases and small G proteins [51-53,72,73]. Phagocytes and the oxidative burst are part of the ancient innate immune system in terms of evolution, and represent the most important barrier for microbes invading the body. NADPH oxidases are enzymes whose biological function is electron transport and the generation of reactive oxygen species (ROS), also a very ancient principle in terms of evolution. They are widely distributed in different kingdoms of life and are present in fungi, plants, and animals. The invention of NADPH oxidase enzymes in the early development of life was a success story: there is no evidence of multicellular life without these enzymes [74]. Thus, it could be possible that the gravitational conditions on Earth were one of the requirements and conditions for the development of the molecular machinery of oxidative burst reaction.

## Abbreviations

AUC: Area under curve (integral); LPS: Lipopolysaccharide; MuSIC: Multi sample incubatinon centrifuge; NADPH oxidase: Nicotinamide adenine dinucleotide phosphate oxidase; NBT: Nitro blue tetrazolium chloride; PMT: Photomultiplier tube; PMN: Polymorphonuclear leukocytes; ROS: Reactive oxygen species; SAHC: Short arm human centrifuge.

## Competing interests

The authors declare that they have no competing interests.

## Authors’ contribution

OU, BH, AA and KS developed the study idea, concept and the overall study design in addition to planning, coordinating and supervising the study. KS, AA and OU wrote and edited the manuscript. JS, SB, MW, RH and WK contributed to the manuscript. AA developed the PMT clinostat. AA and KS performed the experiments during the parabolic flight campaigns and the clinostat experiments. SB carried out the SAHC hypergravity experiments, RH, MW and WK supervised the SAHC experiments. JS contributed to the clinostat studies. All authors read and approved the final manuscript.
